# *N*-(1-Allyl-1*H*-indazol-5-yl)-4-meth­oxy­benzene­sulfonamide hemihydrate

**DOI:** 10.1107/S1600536813025543

**Published:** 2013-09-28

**Authors:** Hakima Chicha, El Mostapha Rakib, Detlef Geffken, Mohamed Saadi, Lahcen El Ammari

**Affiliations:** a Laboratoire de Chimie Organique et Analytique, Université Sultan Moulay Slimane, Faculté des Sciences et Techniques, Béni-Mellal, BP 523, Morocco; b Department of Pharmaceutical Chemistry, Institute of Pharmacy, University of Hamburg, Hamburg, Germany; c Laboratoire de Chimie du Solide Appliquée, Faculté des Sciences, Université Mohammed V-Agdal, Avenue Ibn Battouta, BP. 1014, Rabat, Morocco

## Abstract

In the title compound, C_17_H_17_N_3_O_3_^.^0.5H_2_O, the indazole system makes a dihedral angle of 46.19 (8)° with the plane through the benzene ring and is nearly perpendicular to the allyl group, as indicated by the dihedral angle of 81.2 (3)°. In the crystal, the water mol­ecule, disordered over two sites related by an inversion center, forms O—H⋯N bridges between indazole N atoms of two sulfonamide mol­ecules. It is also connected *via* N—H⋯O inter­action to the third sulfonamide mol­ecule; however, due to the water mol­ecule disorder, only every second mol­ecule of sulfonamide participates in this inter­action. This missing inter­action results in a slight disorder of the sulfonamide S,O and N atoms which are split over two sites with half occupancy. With the help of C–H⋯O hydrogen bonds, the mol­ecules are further connected into a three-dimensional network.

## Related literature

For the pharmacological activity of sulfonamides, see: Bouissane *et al.* (2006 ▶); Supuran & Scozzafava (2003 ▶); Smith & Jones (2008 ▶); Scozzafava *et al.* (2003 ▶). For their anti­proliferative activity, see: Abbassi *et al.* (2012 ▶, 2013 ▶).
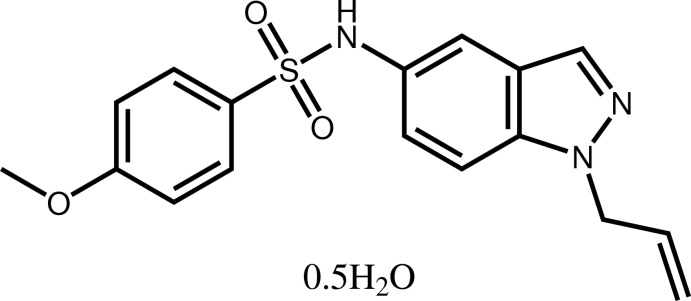


## Experimental

### 

#### Crystal data

2C_17_H_17_N_3_O_3_S·H_2_O
*M* *_r_* = 704.81
Monoclinic, 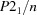

*a* = 8.2099 (7) Å
*b* = 13.8928 (12) Å
*c* = 15.0495 (14) Å
β = 92.327 (3)°
*V* = 1715.1 (3) Å^3^
*Z* = 2
Mo *K*α radiation
μ = 0.21 mm^−1^
*T* = 296 K
0.38 × 0.36 × 0.25 mm

#### Data collection

Bruker X8 APEX diffractometer
Absorption correction: multi-scan (*SADABS*; Sheldrick, 2003 ▶) *T*_min_ = 0.693, *T*_max_ = 0.747
19356 measured reflections
4088 independent reflections
2737 reflections with *I* > 2σ(*I*)
*R*_int_ = 0.031

#### Refinement

*R*[*F* ^2^ > 2σ(*F* ^2^)] = 0.042
*wR*(*F* ^2^) = 0.119
*S* = 1.01
4088 reflections
266 parameters
2 restraints
H-atom parameters constrained
Δρ_max_ = 0.15 e Å^−3^
Δρ_min_ = −0.17 e Å^−3^

### 

Data collection: *APEX2* (Bruker, 2009 ▶); cell refinement: *SAINT* (Bruker, 2009 ▶); data reduction: *SAINT*; program(s) used to solve structure: *SHELXS97* (Sheldrick, 2008 ▶); program(s) used to refine structure: *SHELXL97* (Sheldrick, 2008 ▶); molecular graphics: *ORTEP-3 for Windows* (Farrugia, 2012 ▶); software used to prepare material for publication: *PLATON* (Spek, 2009 ▶) and *publCIF* (Westrip, 2010 ▶).

## Supplementary Material

Crystal structure: contains datablock(s) I. DOI: 10.1107/S1600536813025543/gk2590sup1.cif

Structure factors: contains datablock(s) I. DOI: 10.1107/S1600536813025543/gk2590Isup2.hkl

Supplementary material file. DOI: 10.1107/S1600536813025543/gk2590Isup3.cml

Additional supplementary materials:  crystallographic information; 3D view; checkCIF report

## Figures and Tables

**Table 1:** Hydrogen-bond geometry (Å, °)

*D*—H⋯*A*	*D*—H	H⋯*A*	*D*⋯*A*	*D*—H⋯*A*
N1—H1⋯O4	0.89	1.93	2.802 (6)	165
C3—H3⋯O1′^i^	0.93	2.50	3.237 (9)	136
C8—H8*A*⋯O1′^i^	0.97	2.25	3.202 (4)	168
C7—H7⋯O2^ii^	0.93	2.63	3.447 (7)	147
O4—H4⋯N2^iii^	0.86	2.02	2.748 (3)	142
O4—H4′⋯N2^iv^	0.86	2.29	3.082 (3)	152

Symmetry codes: (i) 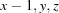; (ii) 
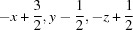
; (iii) 
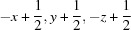
; (iv) 
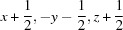
.

**Fig. 1.**
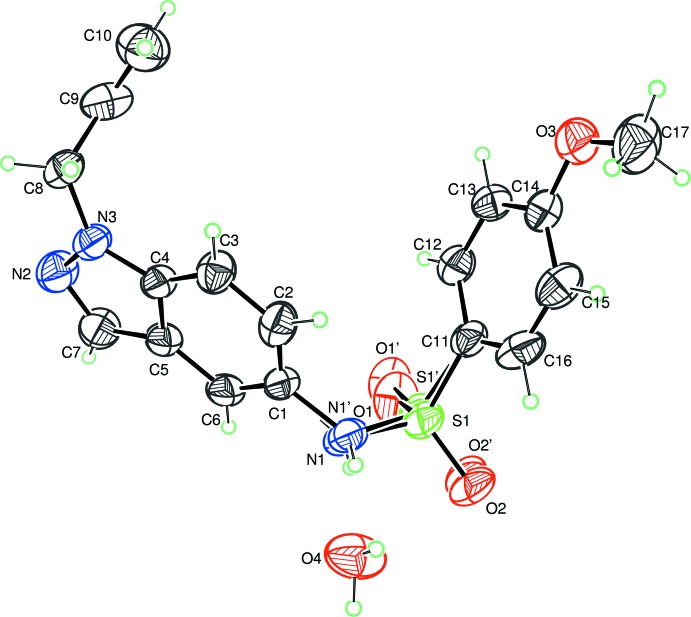
: Molecular structure of the title compound with the atom-labelling scheme. Displacement ellipsoids are drawn at the 50% probability level. H atoms are represented as small circles.

**Fig. 2.**
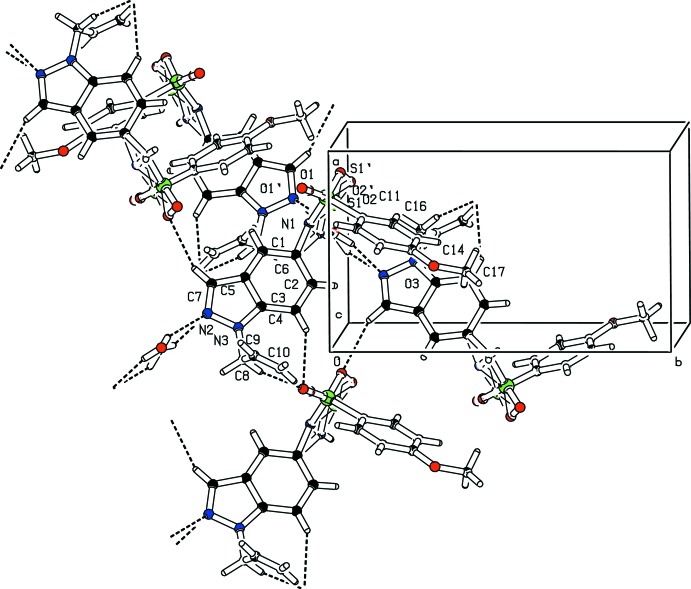
: Partial crystal packing for the title compound showing hydrogen bonds as dashed lines.

## supplementary crystallographic information

### 1. Comment

Sulfonamides are an important class of compounds which are widely used in the design of diverse classes of drug candidates (Supuran & Scozzafava, 2003; Smith & Jones 2008; Scozzafava *et al.*, 2003). Recently, some *N*-[7(6)-indazolyl]arylsulfonamides prepared by our research group showed important antiproliferative activity against some human and murine cell lines. The present structure is a continuation of the investigation of the sulfonamide derivatives published recently by our team (Abbassi *et al.*, 2012; Bouissane *et al.*, 2006; Abbassi *et al.*, 2013).

In this structure, the sulfonamide N1, S1, O1 and O2 atoms are splitted over two sites. They were refined with the occupancy factor of 0.5 as their disorder is related to the disorder of the water molecule: the water molecule is disordered over two sites related by an inversion center.

The molecule of the *N*-(1-Allyl-1*H*-indazol-5-yl)-4-methoxybenzenesulfonamide is built up from the fused five- and six-membered rings (N2 N3 C1—C7) linked to the benzenesulfonamide group as shown in Fig.1. Moreover, the dihedral angle between the indazole system and the plan through the atoms forming the benzene ring (C9—C14) is of 46.19 (8)°. The allyl group is nearly perpendicular to the indazole rings as indicated by the dihedral angle of 81.2 (3)°.

In the crystal, the water molecule acts as a bridge between two molecules through O–H···N hydrogen bonds and every second sulfonamide molecule is involved in N-H···O interaction with the water O atom. The molecules are also interconnected by C–H···O hydrogen bonds forming a three-dimensional network (Fig.2 and Table 1).

### 2. Experimental

A mixture of 1-allyl-5-nitroindazole (1.22 mmol) and anhydrous SnCl_2_ (1.1 g, 6.1 mmol) in 25 ml of absolute ethanol was heated at 333 K for 6 h. After reduction, the starting material disappeared, and the solution was allowed to cool down. The pH was made slightly basic (pH 7–8) by addition of 5% aqueous potassium bicarbonate before extraction with ethyl acetate. The organic phase was washed with brine and dried over magnesium sulfate. The solvent was removed to afford the amine, which was immediately dissolved in pyridine (5 ml) and then reacted with 4-methoxybenzenesulfonyl chloride (1.25 mmol) at room temperature for 24 h. After the reaction mixture was concentrated in vacuo, the resulting residue was purified by flash chromatography (eluted with ethyl acetate: hexane 1:9). The title compound was recrystallized from ethanol (m.p. 370 K, yield: 78%).

### 3. Refinement

H atoms were located in a difference map and were refined as riding with the distance constraints: C–H = 0.93-0.97 Å, O–H = 0.86 Å and N–H = 0.89 Å and with *U*_iso_(H) = 1.2 *U*_eq_ (aromatic, OH, NH) and *U*_iso_(H) = 1.5 *U*_eq_ for methyl group. The N1, S1, O1 and O2 atoms are splitted over two sites with the occupancy factor of 0.5. Restraints were imposed on S1—O1 and S1'—O1' distances 1.446 (1) Å. The occupancy factor of the disordered around inversion center water molecule is 0.5.

### Crystal data

**Table:**

2C_17_H_17_N_3_O_3_S·H_2_O	*F*(000) = 740
*M**_r_* = 704.81	*D*_x_ = 1.365 Mg m^−^^3^
Monoclinic, *P*2_1_/*n*	Melting point: 370 K
Hall symbol: -P 2yn	Mo *K*α radiation, λ = 0.71073 Å
*a* = 8.2099 (7) Å	Cell parameters from 4088 reflections
*b* = 13.8928 (12) Å	θ = 2.7–27.9°
*c* = 15.0495 (14) Å	µ = 0.21 mm^−^^1^
β = 92.327 (3)°	*T* = 296 K
*V* = 1715.1 (3) Å^3^	Block, colourless
*Z* = 2	0.38 × 0.36 × 0.25 mm

### Data collection

**Table:**

Bruker X8 APEX diffractometer	4088 independent reflections
Radiation source: fine-focus sealed tube	2737 reflections with *I* > 2σ(*I*)
Graphite monochromator	*R*_int_ = 0.031
φ and ω scans	θ_max_ = 27.9°, θ_min_ = 2.7°
Absorption correction: multi-scan (*SADABS*; Sheldrick, 2003)	*h* = −10→10
*T*_min_ = 0.693, *T*_max_ = 0.747	*k* = −17→18
19356 measured reflections	*l* = −19→19

### Refinement

**Table:**

Refinement on *F*^2^	Primary atom site location: structure-invariant direct methods
Least-squares matrix: full	Secondary atom site location: difference Fourier map
*R*[*F*^2^ > 2σ(*F*^2^)] = 0.042	Hydrogen site location: inferred from neighbouring sites
*wR*(*F*^2^) = 0.119	H-atom parameters constrained
*S* = 1.01	*w* = 1/[σ^2^(*F*_o_^2^) + (0.0496*P*)^2^ + 0.3501*P*] where *P* = (*F*_o_^2^ + 2*F*_c_^2^)/3
4088 reflections	(Δ/σ)_max_ < 0.001
266 parameters	Δρ_max_ = 0.15 e Å^−^^3^
2 restraints	Δρ_min_ = −0.17 e Å^−^^3^

### Special details

**Table:**

Geometry. All s.u.'s (except the s.u. in the dihedral angle between two l.s. planes) are estimated using the full covariance matrix. The cell s.u.'s are taken into account individually in the estimation of s.u.'s in distances, angles and torsion angles; correlations between s.u.'s in cell parameters are only used when they are defined by crystal symmetry. An approximate (isotropic) treatment of cell s.u.'s is used for estimating s.u.'s involving l.s. planes.
Refinement. Refinement of *F*^2^ against all reflections. The weighted *R*-factor *wR* and goodness of fit *S* are based on *F*^2^, conventional *R*-factors *R* are based on *F*, with *F* set to zero for negative *F*^2^. The threshold expression of *F*^2^ > σ(*F*^2^) is used only for calculating *R*-factors(gt) *etc*. and is not relevant to the choice of reflections for refinement. *R*-factors based on *F*^2^ are statistically about twice as large as those based on *F*, and *R*- factors based on all data will be even larger.

### Fractional atomic coordinates and isotropic or equivalent isotropic displacement parameters (Å^2^)

**Table:**

	*x*	*y*	*z*	*U*_iso_*/*U*_eq_	Occ. (<1)
C1	0.4495 (2)	−0.11076 (14)	0.23403 (11)	0.0607 (5)	
C2	0.3084 (2)	−0.06191 (13)	0.20204 (12)	0.0602 (4)	
H2	0.2989	0.0038	0.2126	0.072*	
C3	0.18511 (19)	−0.10864 (12)	0.15581 (12)	0.0560 (4)	
H3	0.0918	−0.0764	0.1351	0.067*	
C4	0.20595 (18)	−0.20712 (11)	0.14118 (10)	0.0469 (4)	
C5	0.34564 (19)	−0.25681 (12)	0.17146 (11)	0.0514 (4)	
C6	0.4694 (2)	−0.20697 (14)	0.21927 (11)	0.0618 (5)	
H6	0.5628	−0.2388	0.2404	0.074*	
C7	0.3185 (2)	−0.35243 (13)	0.14334 (14)	0.0680 (5)	
H7	0.3916	−0.4026	0.1542	0.082*	
C8	−0.0440 (2)	−0.25522 (14)	0.04742 (13)	0.0656 (5)	
H8A	−0.1088	−0.2084	0.0781	0.079*	
H8B	−0.1062	−0.3145	0.0425	0.079*	
C9	−0.0116 (3)	−0.21878 (16)	−0.04337 (13)	0.0753 (6)	
H9	0.0561	−0.2554	−0.0781	0.090*	
C10	−0.0701 (3)	−0.14065 (18)	−0.07713 (17)	0.0909 (7)	
H10A	−0.1382	−0.1021	−0.0443	0.109*	
H10B	−0.0443	−0.1225	−0.1344	0.109*	
C11	0.64190 (18)	0.08820 (13)	0.17142 (11)	0.0528 (4)	
C12	0.6036 (2)	0.07706 (13)	0.08101 (11)	0.0572 (4)	
H12	0.6267	0.0194	0.0528	0.069*	
C13	0.5320 (2)	0.15075 (13)	0.03369 (11)	0.0574 (4)	
H13	0.5056	0.1426	−0.0265	0.069*	
C14	0.49834 (19)	0.23733 (12)	0.07447 (12)	0.0536 (4)	
C15	0.5389 (2)	0.24981 (15)	0.16372 (13)	0.0670 (5)	
H15	0.5187	0.3082	0.1914	0.080*	
C16	0.6095 (2)	0.17525 (16)	0.21134 (12)	0.0676 (5)	
H16	0.6358	0.1836	0.2715	0.081*	
C17	0.3917 (3)	0.39644 (15)	0.0586 (2)	0.1045 (9)	
H17A	0.3408	0.4371	0.0140	0.157*	
H17B	0.4915	0.4255	0.0805	0.157*	
H17C	0.3197	0.3884	0.1068	0.157*	
N1	0.5992 (6)	−0.0729 (4)	0.2783 (4)	0.0542 (11)	0.50
H1	0.5661	−0.0447	0.3275	0.065*	0.50
S1	0.7087 (4)	0.0029 (3)	0.2443 (3)	0.0586 (6)	0.50
O1	0.7780 (11)	−0.0690 (6)	0.1876 (6)	0.0787 (17)	0.50
O2	0.8022 (7)	0.0461 (5)	0.3174 (4)	0.0836 (15)	0.50
N1'	0.5494 (6)	−0.0450 (4)	0.2905 (4)	0.0524 (11)	0.50
H1'	0.5163	−0.0168	0.3397	0.063*	0.50
S1'	0.7382 (4)	−0.0190 (3)	0.2239 (2)	0.0574 (7)	0.50
O1'	0.7928 (11)	−0.0869 (6)	0.1587 (5)	0.0736 (12)	0.50
O2'	0.8508 (7)	0.0180 (5)	0.2899 (4)	0.0801 (15)	0.50
N2	0.1771 (2)	−0.36215 (11)	0.09978 (11)	0.0683 (4)	
N3	0.10705 (17)	−0.27305 (10)	0.09919 (9)	0.0558 (4)	
O3	0.42555 (16)	0.30521 (9)	0.02113 (9)	0.0731 (4)	
O4	0.5283 (4)	−0.0107 (2)	0.44955 (19)	0.0927 (10)	0.50
H4	0.4728	0.0403	0.4598	0.111*	0.50
H4'	0.5735	−0.0261	0.5001	0.111*	0.50

### Atomic displacement parameters (Å^2^)

**Table:**

	*U*^11^	*U*^22^	*U*^33^	*U*^12^	*U*^13^	*U*^23^
C1	0.0617 (10)	0.0779 (12)	0.0414 (9)	−0.0260 (9)	−0.0095 (7)	0.0098 (8)
C2	0.0597 (10)	0.0590 (10)	0.0623 (11)	−0.0108 (8)	0.0071 (8)	−0.0109 (9)
C3	0.0428 (8)	0.0562 (10)	0.0689 (11)	0.0012 (7)	0.0005 (7)	−0.0025 (8)
C4	0.0431 (8)	0.0524 (9)	0.0447 (8)	−0.0025 (6)	−0.0032 (6)	0.0017 (7)
C5	0.0479 (8)	0.0552 (10)	0.0507 (9)	0.0003 (7)	−0.0046 (7)	0.0098 (7)
C6	0.0507 (9)	0.0754 (12)	0.0581 (10)	−0.0083 (8)	−0.0146 (8)	0.0235 (9)
C7	0.0651 (11)	0.0545 (11)	0.0831 (13)	0.0084 (9)	−0.0107 (10)	0.0057 (9)
C8	0.0545 (10)	0.0683 (12)	0.0723 (12)	−0.0088 (8)	−0.0198 (9)	0.0031 (9)
C9	0.0764 (13)	0.0858 (14)	0.0621 (12)	0.0144 (11)	−0.0179 (10)	−0.0080 (11)
C10	0.0900 (16)	0.0918 (17)	0.0903 (16)	0.0097 (13)	−0.0014 (13)	0.0144 (13)
C11	0.0391 (8)	0.0661 (10)	0.0526 (9)	−0.0119 (7)	−0.0045 (7)	−0.0006 (8)
C12	0.0607 (10)	0.0550 (10)	0.0557 (10)	−0.0045 (8)	−0.0002 (8)	−0.0104 (8)
C13	0.0674 (10)	0.0614 (11)	0.0430 (9)	−0.0061 (8)	−0.0028 (8)	−0.0060 (8)
C14	0.0465 (8)	0.0547 (10)	0.0595 (10)	−0.0072 (7)	−0.0009 (7)	−0.0052 (8)
C15	0.0658 (11)	0.0704 (12)	0.0643 (12)	0.0015 (9)	−0.0020 (9)	−0.0259 (10)
C16	0.0607 (11)	0.0954 (15)	0.0461 (10)	−0.0057 (10)	−0.0061 (8)	−0.0158 (10)
C17	0.1018 (18)	0.0579 (13)	0.151 (2)	0.0092 (12)	−0.0287 (17)	−0.0145 (14)
N1	0.051 (3)	0.064 (3)	0.048 (2)	−0.0047 (19)	−0.009 (2)	−0.0044 (19)
S1	0.0382 (11)	0.0669 (15)	0.0694 (16)	−0.0012 (8)	−0.0146 (9)	0.0034 (9)
O1	0.059 (2)	0.056 (3)	0.122 (6)	0.028 (2)	0.020 (3)	−0.019 (3)
O2	0.069 (3)	0.091 (4)	0.087 (4)	−0.013 (2)	−0.045 (3)	0.003 (2)
N1'	0.050 (3)	0.065 (3)	0.042 (2)	−0.0058 (19)	−0.0044 (18)	−0.0026 (19)
S1'	0.0353 (12)	0.0652 (18)	0.0704 (18)	−0.0059 (9)	−0.0121 (9)	0.0045 (11)
O1'	0.046 (2)	0.064 (3)	0.112 (5)	0.016 (2)	0.014 (3)	−0.017 (2)
O2'	0.056 (3)	0.082 (3)	0.099 (4)	−0.014 (2)	−0.036 (2)	0.007 (2)
N2	0.0745 (10)	0.0528 (9)	0.0764 (11)	0.0032 (7)	−0.0109 (8)	−0.0062 (8)
N3	0.0533 (8)	0.0537 (8)	0.0590 (8)	−0.0026 (6)	−0.0133 (6)	−0.0004 (7)
O3	0.0735 (9)	0.0590 (8)	0.0856 (9)	−0.0007 (6)	−0.0104 (7)	0.0020 (7)
O4	0.133 (3)	0.0734 (18)	0.0708 (17)	0.0370 (18)	−0.0081 (18)	−0.0020 (15)

### Geometric parameters (Å, º)

**Table:**

C1—C6	1.366 (3)	C11—S1'	1.848 (4)
C1—C2	1.411 (3)	C12—C13	1.366 (2)
C1—N1	1.471 (6)	C12—H12	0.9300
C1—N1'	1.474 (6)	C13—C14	1.383 (2)
C2—C3	1.369 (2)	C13—H13	0.9300
C2—H2	0.9300	C14—O3	1.361 (2)
C3—C4	1.397 (2)	C14—C15	1.382 (3)
C3—H3	0.9300	C15—C16	1.374 (3)
C4—N3	1.3623 (19)	C15—H15	0.9300
C4—C5	1.399 (2)	C16—H16	0.9300
C5—C6	1.403 (2)	C17—O3	1.419 (3)
C5—C7	1.409 (2)	C17—H17A	0.9600
C6—H6	0.9300	C17—H17B	0.9600
C7—N2	1.317 (2)	C17—H17C	0.9600
C7—H7	0.9300	N1—S1	1.489 (8)
C8—N3	1.459 (2)	N1—H1	0.8898
C8—C9	1.491 (3)	S1—O2	1.445 (7)
C8—H8A	0.9700	S1—O1	1.4463 (10)
C8—H8B	0.9700	N1'—S1'	1.914 (7)
C9—C10	1.283 (3)	N1'—H1'	0.8898
C9—H9	0.9300	S1'—O2'	1.425 (7)
C10—H10A	0.9300	S1'—O1'	1.4460 (10)
C10—H10B	0.9300	N2—N3	1.365 (2)
C11—C16	1.381 (3)	O4—O4^i^	1.633 (6)
C11—C12	1.393 (2)	O4—H4	0.8600
C11—S1	1.691 (5)	O4—H4'	0.8600
			
C6—C1—C2	121.13 (15)	C12—C13—H13	119.7
C6—C1—N1	108.7 (2)	C14—C13—H13	119.7
C2—C1—N1	129.9 (3)	O3—C14—C15	124.71 (16)
C6—C1—N1'	129.3 (2)	O3—C14—C13	115.57 (15)
C2—C1—N1'	109.2 (2)	C15—C14—C13	119.73 (17)
C3—C2—C1	121.71 (17)	C16—C15—C14	119.50 (17)
C3—C2—H2	119.1	C16—C15—H15	120.3
C1—C2—H2	119.1	C14—C15—H15	120.3
C2—C3—C4	116.86 (16)	C15—C16—C11	121.18 (16)
C2—C3—H3	121.6	C15—C16—H16	119.4
C4—C3—H3	121.6	C11—C16—H16	119.4
N3—C4—C3	131.06 (14)	O3—C17—H17A	109.5
N3—C4—C5	106.54 (14)	O3—C17—H17B	109.5
C3—C4—C5	122.40 (14)	H17A—C17—H17B	109.5
C4—C5—C6	119.36 (16)	O3—C17—H17C	109.5
C4—C5—C7	104.47 (14)	H17A—C17—H17C	109.5
C6—C5—C7	136.17 (16)	H17B—C17—H17C	109.5
C1—C6—C5	118.54 (16)	C1—N1—S1	127.0 (5)
C1—C6—H6	120.7	C1—N1—H1	104.9
C5—C6—H6	120.7	S1—N1—H1	100.6
N2—C7—C5	111.81 (15)	O2—S1—O1	121.8 (5)
N2—C7—H7	124.1	O2—S1—N1	110.0 (5)
C5—C7—H7	124.1	O1—S1—N1	88.4 (5)
N3—C8—C9	111.58 (16)	O2—S1—C11	110.6 (4)
N3—C8—H8A	109.3	O1—S1—C11	103.1 (5)
C9—C8—H8A	109.3	N1—S1—C11	122.3 (3)
N3—C8—H8B	109.3	C1—N1'—S1'	104.9 (3)
C9—C8—H8B	109.3	C1—N1'—H1	132.6
H8A—C8—H8B	108.0	S1'—N1'—H1	110.1
C10—C9—C8	124.9 (2)	C1—N1'—H1'	124.8
C10—C9—H9	117.6	S1'—N1'—H1'	129.3
C8—C9—H9	117.6	O2'—S1'—O1'	119.8 (4)
C9—C10—H10A	120.0	O2'—S1'—C11	105.2 (3)
C9—C10—H10B	120.0	O1'—S1'—C11	112.0 (5)
H10A—C10—H10B	120.0	O2'—S1'—N1'	102.7 (4)
C16—C11—C12	118.85 (17)	O1'—S1'—N1'	120.8 (4)
C16—C11—S1	113.23 (17)	C11—S1'—N1'	92.0 (2)
C12—C11—S1	127.62 (18)	C7—N2—N3	105.84 (14)
C16—C11—S1'	127.33 (16)	C4—N3—N2	111.33 (13)
C12—C11—S1'	113.81 (17)	C4—N3—C8	127.69 (14)
C13—C12—C11	120.08 (16)	N2—N3—C8	120.42 (14)
C13—C12—H12	120.0	C14—O3—C17	118.21 (17)
C11—C12—H12	120.0	H4—O4—H4'	104.9
C12—C13—C14	120.64 (16)		
			
C6—C1—C2—C3	0.6 (3)	C16—C11—S1—O2	−32.4 (4)
N1—C1—C2—C3	174.6 (3)	C12—C11—S1—O2	154.1 (3)
N1'—C1—C2—C3	−172.7 (3)	S1'—C11—S1—O2	124.8 (11)
C1—C2—C3—C4	−0.4 (3)	C16—C11—S1—O1	−164.0 (4)
C2—C3—C4—N3	179.20 (17)	C12—C11—S1—O1	22.4 (5)
C2—C3—C4—C5	−0.3 (3)	S1'—C11—S1—O1	−6.8 (9)
N3—C4—C5—C6	−178.82 (15)	C16—C11—S1—N1	99.6 (3)
C3—C4—C5—C6	0.8 (3)	C12—C11—S1—N1	−74.0 (3)
N3—C4—C5—C7	0.46 (18)	S1'—C11—S1—N1	−103.2 (11)
C3—C4—C5—C7	−179.94 (17)	C6—C1—N1'—S1'	77.0 (4)
C2—C1—C6—C5	−0.1 (3)	C2—C1—N1'—S1'	−110.3 (2)
N1—C1—C6—C5	−175.3 (3)	N1—C1—N1'—S1'	44.8 (10)
N1'—C1—C6—C5	171.8 (3)	C16—C11—S1'—O2'	−32.7 (4)
C4—C5—C6—C1	−0.6 (3)	C12—C11—S1'—O2'	145.7 (3)
C7—C5—C6—C1	−179.5 (2)	S1—C11—S1'—O2'	−59.3 (9)
C4—C5—C7—N2	0.1 (2)	C16—C11—S1'—O1'	−164.4 (4)
C6—C5—C7—N2	179.2 (2)	C12—C11—S1'—O1'	14.0 (5)
N3—C8—C9—C10	−125.3 (2)	S1—C11—S1'—O1'	169.0 (11)
C16—C11—C12—C13	−1.4 (3)	C16—C11—S1'—N1'	71.0 (3)
S1—C11—C12—C13	171.9 (2)	C12—C11—S1'—N1'	−110.6 (2)
S1'—C11—C12—C13	−179.90 (18)	S1—C11—S1'—N1'	44.4 (9)
C11—C12—C13—C14	0.7 (3)	C1—N1'—S1'—O2'	−162.4 (4)
C12—C13—C14—O3	−179.39 (15)	C1—N1'—S1'—O1'	−25.8 (6)
C12—C13—C14—C15	0.6 (3)	C1—N1'—S1'—C11	91.5 (3)
O3—C14—C15—C16	178.76 (17)	C5—C7—N2—N3	−0.7 (2)
C13—C14—C15—C16	−1.3 (3)	C3—C4—N3—N2	179.53 (18)
C14—C15—C16—C11	0.6 (3)	C5—C4—N3—N2	−0.92 (18)
C12—C11—C16—C15	0.7 (3)	C3—C4—N3—C8	8.2 (3)
S1—C11—C16—C15	−173.5 (2)	C5—C4—N3—C8	−172.26 (17)
S1'—C11—C16—C15	179.0 (2)	C7—N2—N3—C4	1.0 (2)
C6—C1—N1—S1	118.9 (3)	C7—N2—N3—C8	173.05 (17)
C2—C1—N1—S1	−55.7 (5)	C9—C8—N3—C4	78.0 (2)
N1'—C1—N1—S1	−86.9 (12)	C9—C8—N3—N2	−92.6 (2)
C1—N1—S1—O2	157.9 (4)	C15—C14—O3—C17	1.5 (3)
C1—N1—S1—O1	−78.7 (5)	C13—C14—O3—C17	−178.45 (18)
C1—N1—S1—C11	25.7 (5)		

Symmetry code: (i) −*x*+1, −*y*, −*z*+1.

### Hydrogen-bond geometry (Å, º)

**Table:**

*D*—H···*A*	*D*—H	H···*A*	*D*···*A*	*D*—H···*A*
N1—H1···O4	0.89	1.93	2.802 (6)	165
C3—H3···O1′^ii^	0.93	2.50	3.237 (9)	136
C8—H8*A*···O1′^ii^	0.97	2.25	3.202 (4)	168
C7—H7···O2^iii^	0.93	2.63	3.447 (7)	147
O4—H4···N2^iv^	0.86	2.02	2.748 (3)	142
O4—H4′···N2^v^	0.86	2.29	3.082 (3)	152

Symmetry codes: (ii) *x*−1, *y*, *z*; (iii) −*x*+3/2, *y*−1/2, −*z*+1/2; (iv) −*x*+1/2, *y*+1/2, −*z*+1/2; (v) *x*+1/2, −*y*−1/2, *z*+1/2.
